# Molecular phylogenetic relationships based on chloroplast genomes of Zingiberaceae species: Insights into evolution and adaptation to extreme environments

**DOI:** 10.3389/fpls.2025.1670568

**Published:** 2025-09-24

**Authors:** Tian Lu, Yebing Yin, Jinglin Luo, Jiao Chen, Yu Wu, Wu Zhang, Yiling Wei, Tao Yuan

**Affiliations:** ^1^ School of Municipal and Environmental Engineering, Shandong Jianzhu University, Jinan, China; ^2^ Key Laboratory of Biodiversity and Environment on the Qinghai-Tibetan Plateau, Ministry of Education, School of Ecology and Environment, Tibet University, Lhasa, China; ^3^ State Key Laboratory of Hybrid Rice, Laboratory of Plant Systematics and Evolutionary Biology, College of Life Sciences, Wuhan University, Wuhan, China; ^4^ Department of Orthopaedics, Yichang Central People’s Hospital, Yichang, China; ^5^ Medical Department, Qiannan National Medical College, Duyun, China

**Keywords:** Zingiberaceae, chloroplast genome, phylogenetic analysis, biogeographic dispersal, positive selection

## Abstract

**Introduction:**

The Zingiberaceae family, which includes numerous economically and medicinally important species, exhibits considerable phylogenetic and genetic diversity. Chloroplast genomes are valuable resources for studying evolutionary relationships, genetic diversity, and adaptive evolution in plants. While many Zingiberaceae chloroplast genomes have been sequenced, the evolutionary mechanisms—including structural variation, codon usage bias, selection pressures, and divergence history—remain to be comprehensively investigated.

**Methods:**

we performed a comparative analysis of 11 newly identified species (Aframomum alboviolaceum, Amomum longipetiolatum, Amomum petaloideum, Amomum velutinum, Cautleya spicata, Cornukaempferia larsenii, Globba atrosanguinea, Globba variabilis, Hedychium aureum, Riedelia arfakensis, and Zingiber citriodorum) and 110 published data from the Zingiberaceae family, including their structure, codon usage, nucleotide polymorphisms, divergence time, and selection pressures.

**Results:**

The chloroplast genomes of Zingiberaceae species exhibited a highly conserved structure with no significant expansion or contraction during diversification. Analysis revealed four hypervariable protein-coding genes (atpH, rpl32, ndhA, and ycf1) and one intergenic region (psac-ndhE), which are proposed as potential molecular markers for future phylogeographic and population genetic studies. Codon usage bias was found to be predominantly shaped by natural selection. Phylogenetic analysis strongly supported the division of Zingiberaceae into two primary subfamilies (Alpinioideae and Zingiberoideae) and clarified key relationships, revealing that Globba is more closely related to Curcuma than to Hedychium, and Hedychium is more closely related to the Pommereschea-Rhynchanthus clade than to Cautleya. Divergence time estimation indicated two rapid diversification events within Zingiberoideae, coinciding with the rapid uplift of the Tibetan Plateau and a Late Miocene cooling event linked to declining CO₂ levels. Ancestral range reconstruction suggested an African origin during the Cretaceous period, followed by dispersal to Southeast Asia and India. Selection pressure analysis showed that most protein-coding genes are under negative selection. In contrast, the ycf2 gene was found to be under relaxed selection. Furthermore, two genes (matK and ndhB) were identified to be under positive selection in high-altitude species of Roscoea, suggesting a role in adaptation to alpine environments.

**Discussion:**

This study provides a comprehensive genomic analysis of the Zingiberaceae family, highlighting the conserved nature of chloroplast genome structure despite extensive diversification. The identified mutation hotspots present valuable tools for developing high-resolution markers for species identification and biogeographic studies. The phylogenetic results resolve longstanding uncertainties in the relationships among key genera. The inferred divergence times and ancestral range suggest that the evolutionary history of Zingiberaceae was significantly influenced by major geological and climatic events, notably the uplift of the Tibetan Plateau and global cooling in the Late Miocene. The prevalence of negative/purifying selection across most genes indicates strong evolutionary constraints to maintain core photosynthetic functions. The discovery of positively selected genes in high-altitude Roscoea species provides insights into adaptive evolution to environmental stressors. These findings offer foundational knowledge for future efforts in crop improvement, species identification, and the conservation of genetic diversity within the Zingiberaceae family.

## Introduction

The family of Zingiberaceae Martinov, part of the Zingiberales order, is a taxonomically complex group known for its significant medicinal, culinary, and horticultural value. This family comprises approximately 1,500 species, organized into two subfamilies and 52 genera. China is home to 21 genera and about 210 species, predominantly found in the tropical and subtropical regions of southern China and Southeast Asia (Soltis et al., 2000; Kress et al., 2002; Kanlayanapaphon and Newman, 2003; Liu, 2003; Branney, 2005; Wu et al., 2016). Many species within Zingiberaceae are characterized by their vibrant bracts and flowers, making them popular choices in landscaping and floral arrangements (Wang et al., 2023; Wang et al., 2024). Notable examples include *Zingiber montanum* (J. König) Link ex A. Dietr. and *Zingiber citriodorum* Theilade & Mood (Branney, 2005; Gao, 2006; Wu et al., 2016). Additionally, the family includes a variety of economically and medicinally important crops, such as *Zingiber mioga* (Thunb.) Roscoe, *Zingiber zerumbet* (L.) Roscoe ex Sm., *Zingiber officinale* Roscoe, *Alpinia galanga* (L.) Willd., and *Alpinia kwangsiensis* T. L. Wu & S. J. Chen (Gao, 2006; Sasidharan and Nirmala, 2010; Banerjee et al., 2011; Prasad and Tyagi, 2015). In traditional Chinese medicine, the rhizomes of *A. kwangsiensis* are utilized for the treatment of abdominal pain, vomiting, and traumatic injuries (Medicinal Materials Company in Yunnan Province, 1993; Wu et al., 2015). *Alpinia zerumbet* (Pers.) B. L. Burtt & R. M. Sm. exhibits important physiological and pharmacological properties, including antioxidant and antimicrobial effects, anxiolytic properties, and the promotion of osteoblast differentiation activity (Kumagai et al., 2016; Xuan et al., 2016; Castro et al., 2018). *A. galanga* is utilized as a remedy for various conditions, serving as an antifungal, antimicrobial, anti-inflammatory, and antioxidant agent, as well as in the treatment of osteoarthritis (Chouni and Paul, 2018).

Although previous studies have investigated individual genera (e.g., *Zingiber* Boehm., *Curcuma* L., *Stahlianthus* Kuntze, and *Alpinia* Roxb.), large-scale phylogenetic reconstructions of Zingiberaceae remain fragmented (Li et al., 2019; Liang et al., 2020; Li et al., 2020b). At the same time, extensive intermediate hybridization and morphological similarities contribute to the phylogenetic complexity within Zingiberaceae. Taking the genera *Curcuma* and *Amomum* Roxb. as examples, *Curcuma flaviflora* S. Q. Tong exhibits the distinct morphological features of *Curcuma*, including flowers with funnel-shaped corollas, petaloid lateral staminodes, lobes that are oblong and ovate, and a base adnate to the labellum and filament. However, a phylogenetic analysis based on chloroplast genomes showed that *C. flaviflora* clustered with *Zingiber* (Liang et al., 2020). Given the overlap in their distribution ranges, introgressive hybridization may be widespread, leading to complex affinities between *Curcuma* and *Zingiber*. Furthermore, *Amomum* exhibits significant differences in morphology, habit, inflorescence, and capsule structure, with previous studies indicating that *Amomum* belongs to a polyphyletic lineage (Kress et al., 2002; Xia et al., 2004). Thus, *Curcuma* may have complex affinities with *Amomum* and potentially with plants of the Zingiberaceae family. Additional samples and DNA data are required to confirm their phylogenetic relationships. Overall, these previous studies have succeeded in clarifying the phylogenetic relationships of some Zingiberaceae species (Kress et al., 2002; Kanlayanapaphon and Newman, 2003; Xia et al., 2004; Liang et al., 2020; Li et al., 2020b). However, the phylogenetic relationships and taxonomic classification of Zingiberaceae pose significant challenges due to the lack of reliable reference genomes and extensive introgressive hybridization. Many species of Zingiberaceae are common or even dominant understory herbs in the native vegetation of the tropics, whereas some thrive in specific habitats, such as *Hedychium villosum* Wall. in limestone areas, where it plays a crucial role in maintaining forest ecosystem stability (Liu, 2003). Additionally, the genus *Roscoea* Sm. primarily inhabits high-altitude regions like the Yunnan–Guizhou Plateau and the Tibetan Plateau, where it contributes significantly to plateau ecosystem maintenance (Liu, 2003). Most Zingiberaceae species possess economic value, leading to extensive anthropogenic harvesting. Consequently, native vegetation is shrinking and being destroyed, posing threats to the survival of many Zingiberaceae plants. Several species, such as *Etlingera yunnanensis* (T. L. Wu & S. J. Chen) R. M. Sm., *Curcuma exigua* N. Liu, *Siliquamomum tonkinense* Baill., *Amomum hainanense* Y. S. Ye, J. P. Liao & P. Zou, and *A. petaloideum* (S. Q. Tong) T. L. Wu, are endangered and listed in the national list of wild plants under key protection (State Forestry and Grassland Administration of the People’s Republic of China and Ministry of Agriculture and Rural Development of the People’s Republic of China, 2021). *C. exigua* may even have disappeared from its native range. Another example is *S. tonkinense*, distributed in southeastern Yunnan, which has become challenging to find due to forest destruction and loss of shade and humidity. Therefore, we included 121 species across 20 major genera, specifically targeting controversial nodes (e.g., *Wurfbainia* Giseke-*Alpinia-Amomum*) to help clarify unresolved evolutionary relationships and develop molecular markers with enhanced resolution based on them. This will establish a molecular foundation for conserving Zingiberaceae plant diversity.

As one of the pivotal organelles in plants, chloroplasts play an indispensable role in numerous vital biochemical processes and photosynthesis (Neuhaus and Emes, 2000). Unlike nuclear genes in hermaphrodites, chloroplast DNA is inherited maternally, typically lacks recombination in most angiosperms, and displays a low mutation rate (Palmer et al., 1988). Consequently, chloroplast genomic data has become a potent and straightforward strategy for analyzing evolutionary relationships in plants. In contrast to relying solely on one or a few specific DNA fragments, which often results in insufficient evidence, incomplete conclusions, and heightened confusion, the complete chloroplast genomes offer a more robust solution for species identification and phylogeny reconstruction (Rogalski et al., 2015). The advancement of sequencing technologies has led to a significant increase in the number of chloroplast genomes. Unfortunately, publicly available data on chloroplast genomes within the Zingiberaceae family remain scarce. This scarcity of complete chloroplast genomic data severely hampers phylogenetic analysis and genetic breeding studies within the Zingiberaceae family.

Our newly generated chloroplast genome data cover 11 species in 8 genera, which represent distinct phylogenetic clades within Zingiberaceae. By including a wide range of taxonomically distinct genera, we aim to elucidate phylogenetic patterns within the family. Some genera exhibit clear differences in ecological niche (e.g., *Roscoea* in alpine habitats versus *Curcuma* in tropical understories). By combining comparative analyses with a broad sampling strategy, we assessed the influence of environmental factors on the adaptive evolution of species in different genera. Previous studies on phylogenetic analysis focused predominantly on individual genera, including *Zingiber* and *Curcuma*. Our cross-generic comparison provides critical insights into the universality/specificity of reported mechanisms within Zingiberaceae. Our goals were as follows:

1. to deduce phylogenetic relationships in Zingiberaceae through a comprehensive chloroplast genomes;
2. to identify highly polymorphic regions and high-resolution molecular markers for Zingiberaceae, providing a theoretical basis for species identification and conservation; and
3. to explore the adaptive evolution of the genus *Roscoea* in high-altitude environments through chloroplast genome analysis.

## Results

### Characterization of the chloroplast genomes

**Figure 1:**
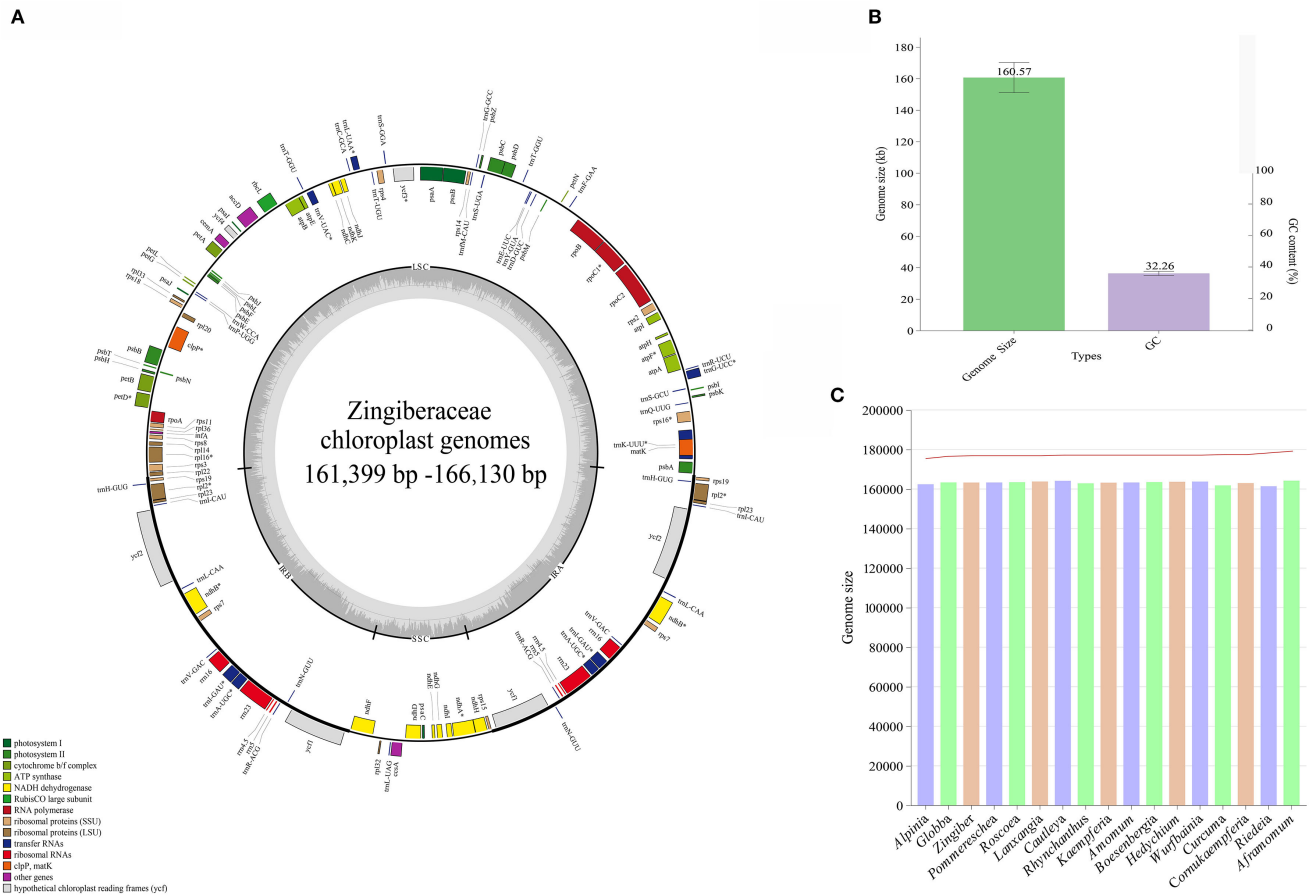
Genomic characterization statistics of 11 assembled chloroplast genomes. **(A)** Gene map of the Zingiberoideae chloroplast genomes. Genes inside and outside the circle are transcribed clockwise and counterclockwise, respectively. Genes belonging to different functional groups are indicated by different colors. **(B)** Mean chloroplast genomes length and GC content of Zingiberaceae species. **(C)** The chloroplast genomes length and GC content statistics for 17 genera.

The 11 *de novo* assembled chloroplast genomes of Zingiberaceae species ranged from 161,399 bp to 166,130 bp (**Table 1** and **Figure 1**), all of which exhibited the typical quadripartite structure (Wu et al., 2017; Liang et al., 2020; Wang et al., 2024). These genomes contained 128–132 functional genes (86–87 protein-coding genes (PCGs), 35–38 tRNAs, and 8 rRNAs; **Table 2**), consistent with published Zingiberaceae data (Li et al., 2020b; Zhang et al., 2021; Wang et al., 2024). Our comprehensive analysis of 113 genomes (11 newly assembled and 102 from NCBI) revealed conserved genomic features within the family: an average size of 160,570 bp (with *Zingiber* sp*ectabile* Griff. as the smallest at 155,890 bp and *Aframomum alboviolaceum* (Ridl.) K.Schum. as the largest at 166,130 bp) and GC content of 36.26% (with *Alpinia* as the lowest at 35.5% and *Riedelia* as the highest at 36.5%). Among the assembled Zingiberaceae chloroplast genomes, the smallest was that of *Z.* sp*ectabile* (155,890 bp), whereas the largest belonged to *A. alboviolaceum* (166,130 bp) (**Supplementary 2**). Among the 17 genera (excluded *Siamanthus* K. Larsen & Mood and *Renealmia* due to incomplete chloroplast genomes), *Cautleya* (Royle ex Benth. & Hook. f.) Hook. f. exhibited the smallest mean chloroplast genome length (164,140 bp), whereas *Aframomum* K. Schum. displayed the largest length (166,130 bp). *Alpinia* had the lowest GC content (35.5%), whereas *Riedelia* had the highest (36.5%), as shown in **Figure 1**.

**Table 1:** Genes present in 11 assembled chloroplast genomes.

Category	Group genes	Name of genes
Transcription and translation	Large subunit of ribosome (LSU)	*rpl2*(x2)*, rpl14, rpl16, rpl20, rpl22, rpl23*(x2)*, rpl32, rpl33, rpl36*
	Small subunit of ribosome (SSU)	*rps2, rps3, rps4, rps7* (x 2)*, rps8, rps11, rps14, rps15, rps16, rps18, rps19*(x2)
	RNA polymerase	*rpoA, rpoB, rpoC1, rpoC2*
	Translational initiation factor	*InfA*
	rRNA genes	*rrn4.5* (x 2)*, rrn5* (x 2)*, rrn16* (x 2)*, rrn23* (x 2)
	tRNA genes	*trnA-UGC* (x 3)*, trnC-GCA, trnD-GUC, trnE-UUC, trnF-GAA, trnfM-CAU, trnG-GCC, trnG-UCC, trnH-GUG (x2), trnI-CAU* (x2)*, trnI-GAU* (x 2)*, trnK-UUU, trnL-CAA* (x 2)*, trnL-UAA, trnL-UAG, trnM-CAU, trnNGUU*(x 2)*, trnP-UGG, trnQ-UUG, trnR-ACG* (x 2)*, trnR-UCU, trnS-GCU* (x2)*, trnS-GGA, trnS-UGA, trnTGGU, trnT-UGU, trnV-GAC* (x 2)*, trnV-UAC, trnW-CCA,trnY-GUA*
Photosynthesis	Photosystem I	*psaA, psaB, psaC, psaI, psaJ*
	Photosystem II	*psbA, psbB, psbC, psbD, psbE, psbF, psbH, psbI, psbJ, psbK, psbL, psbM, psbN, psbT, psbZ*
	NADH oxidoreductase	*ndhA, ndhB* (x 2)*, ndhC, ndhD, ndhE, ndhF, ndhG, ndhH, ndhI, ndhJ, ndhK*
	Cytochrome b6/f complex	*petA, petB, petD, petG, petL, petN*
	ATP synthase	*atpA, atpB, atpE, atpF, atpH, atpI*
	RubiscoCO large subunit	*rbcL*
	ATP-dependent protease subunit gene	*clpP*
Other genes	Maturase	*matK*
	Envelop membrane protein	*cemA*
	Subunit Acetyl- CoA-Carboxylate	*accD*
	c-type cytochrome synthesis gene	*ccsA*
Unknown	Conserved Open reading frames	*ycf1* (x 2)*, ycf2* (x 2)*, ycf3, ycf4*

**Table 2:** The summary of 11 Complete chloroplast genomes for Zingiberaceae crops.

Types	*A. alboviolaceum*	*A. longipetiolatum*	*A. petaloideum*	*A. velutinum*	*C.* sp*icata*	*C. larsenii*	*G. atrosanguinea*	*G. variabilis*	*H. aureum*	*R. arfakensis*	*Z. citriodorum*
GenBank number	PP542015	PP542016	PP542017	PP542018	PP542019	PP542020	PP542021	PP542022	PP542023	PP542024	PP542025
Total length (bp)	166130	163269	163609	163311	164450	162593	161896	165482	163395	161399	165488
Total genes	133	133	133	133	133	132	133	132	132	129	133
Protein-coding genes	87	87	87	87	87	87	87	87	87	86	87
tRNA genes	38	38	38	38	38	37	38	37	37	35	38
rRNA genes	8	8	8	8	8	8	8	8	8	8	8
GC content (%)	35.8	36.1	36.1	36.1	36.0	36.3	35.8	36.2	36.2	36.5	35.8

### Analyses of codon usage

**Figure 2:**
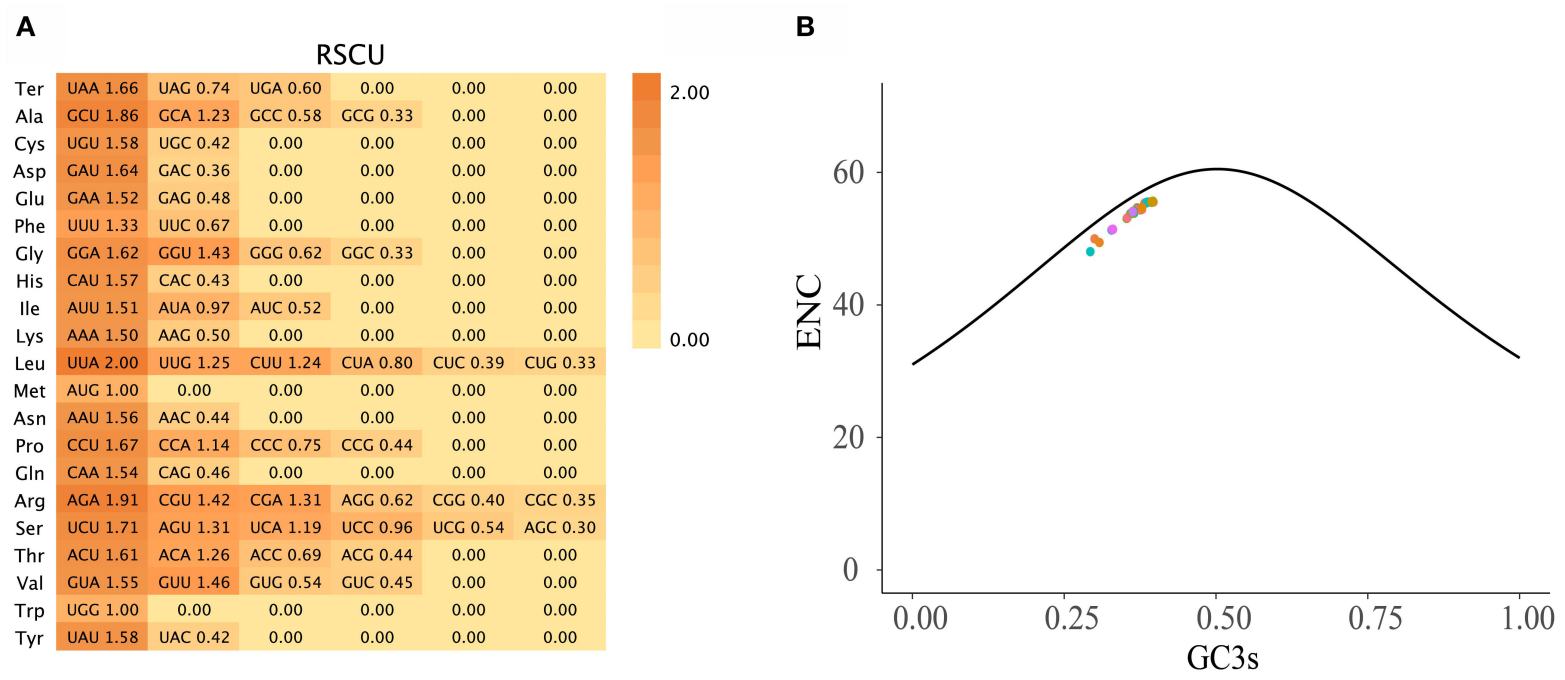
**(A)** The RSCU values in all PCGs in the 121 Zingiberoideae chloroplast genomes. **(B)** ENc plotted against GC3s based on PCGs of 121 Zingiberoideae chloroplast genomes. The solid line indicates the expected curve of positions of genes when the codon usage is merely determined by the GC3s composition.

Codon usage analysis of 78 PCGs across 121 Zingiberoideae chloroplast genomes revealed distinct patterns in synonymous codon usage (**Figure 2**). The total codon count per chloroplast genome ranged from 1,909 to 120,956 (**Supplementary 3**), with phenylalanine (Phe), lysine (Lys), glutamic acid (Glu), and isoleucine (Ile) representing the most abundant amino acids. As expected, methionine (AUG) and tryptophan (UGG) codons showed no usage bias (relative synonymous codon usage (RSCU) = 1.00), consistent with previous studies (Wu et al., 2017; Liang et al., 2020; Zhang et al., 2021). Among the 30 codons displaying preferential usage (RSCU > 1), UUA (leucine) and AGA (arginine) showed the strongest bias, mirroring patterns observed in *Z. officinale*, *A. galanga*, and *A. kwangsiensis* (Wu et al., 2017; Liang et al., 2020; Zhang et al., 2021). The effective number of codons (ENc) ranged from 48.04 (*Pommereschea lackneri* Wittm.) to 55.66 (*Siamanthus siliquosus* K. Larsen et Mood), with no PCGs exhibiting values below 35 (**Figure 2**); thus, these Zingiberoideae PCGs of chloroplast genomes did not exhibit a strong codon preference. This phenomenon likely reflects diminished selection pressure for PCGs in chloroplasts. Meanwhile, the AT-rich chloroplast genome (**Supplementary 3**) predisposes synonymous sites to AT-biased mutations (e.g., UUA-Leu RSCU = 1.6636 and GCU-Ala RSCU = 1.8566), which may override the selection for GC-ending optimal codons.

### Comparative analysis of the chloroplast genomes

The chloroplast genomes of 17 Zingiberoideae species from 17 genera were analyzed through multiple comparisons using mVISTA software, with the *Z. officinale* genome sequence as a reference. The results indicated greater divergence in the LSC and SSC regions compared with the two IR regions (**Supplementary Figure S2**), consistent with previous studies (Cui et al., 2019; Li et al., 2020b; Yu et al., 2023). Non-coding regions exhibited higher divergence levels than coding regions. Approximately 13 highly divergent regions were identified by mVISTA, mainly distributed in non-coding regions, namely, *rps16-trnQ-UUG, psbK-psbI, atpI-atpH, rpoB-psbD, psaA-rps4, rps4-ndhJ, ndhK-atpE, rbcL-accD, accD-psaI, petA-psbJ, psbE-petG, ndhF-rpl32*, and *rpl32-trnL-UAG*. A total of 12 genes, namely, *trnK-UUU, rps16, trnG-UCC, atpF, rpoC1, ycf3, trnV-UAC, clpP, petB, rpl16, ndhA*, and *ycf1*, were also identified Some of these regions, such as *accD-psaI, atpI-atpH, rbcL-accD*, and *ycf1* genes, have been previously observed in the chloroplast genomes of other Zingiberaceae plants (Zhang et al., 2016; Wu et al., 2017; Cui et al., 2019; Li et al., 2020b).

**Figure 3:**
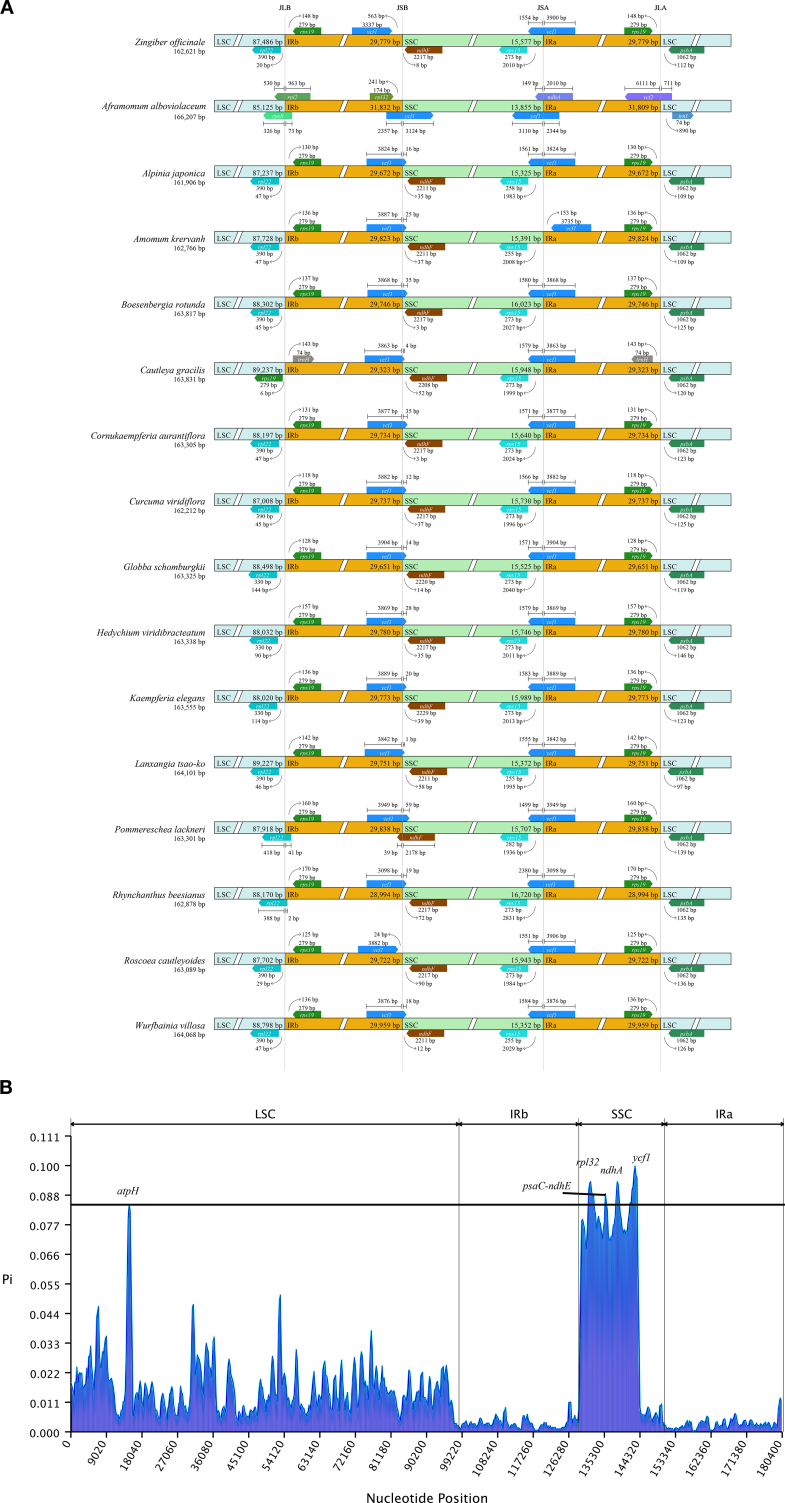
**(A)** Comparison of the junctions between the LSC, SSC and IR regions among 17 Zingiberaceae chloroplast genomes. **(B)** Sliding window analysis of 17 Zingiberaceae chloroplast genomes.

As depicted in **Figure 3**, we also investigated the binding regions of IR/LSC and IR/SSC. The genes *rpl22*, *ndhF*, *ycf1*, and *rps19* were found at the junctions of the LSC/IRb, IRb/SSC, SSC/IRa, and IRa/LSC boundaries, respectively. Noteworthy observations included the relocation of the *ndhF* gene to the IRb region in *P. lackneri*, the shift in the *ycf1* gene to the IRa region in *A. krervanh* Pierre ex Gagnep., the movement of the *rps19* gene to the LSC region in *C. gracilis* (Sm.) Dandy, and the transfer of the *rpl22* gene to the IRb region in *Rhynchanthus beesianus* W. W. Sm. Overall, the chloroplast structure of Zingiberoideae species remained consistent. Additionally, nucleotide diversity (Pi) values were analyzed by DnaSP software to assess the divergence level within different regions among the 17 Zingiberoideae chloroplast genomes from 17 genera. The results showed that Pi values for each gene ranged from 0.00022 to 0.09952 (**Supplementary 4**). Among the 78 PCGs (CDS regions), Pi values ranged from 0.00089 (*rps12*) to 0.09952 (*rps19*), with an average value of 0.00182. For the non-coding regions, Pi values ranged from 0.0022 (*rps12*-exon2-*rps12*-exon3) to 0.09362 (*ndhA*-exon2-*ndhA*-exon1), with an average of 0.01844. These results also demonstrated that the average Pi value in the non-coding regions was more than 10 times higher than that in the coding regions. Four PCGs regions (*atpH*, *rpl32*, *ndhA*, and *ycf1*) and one intergenic region (*psaC*-*ndhE*) exhibited significant differences (Pi>0.085), making them suitable DNA markers for species identification in Zingiberoideae plants.

To elucidate evolutionary relationships among Zingiberoideae species at the genome level, we conducted collinearity block analysis of their chloroplast genomes. The results demonstrated remarkable structural conservation, with Mauve alignment revealing four conserved locally collinear blocks across all 17 genera examined (**Supplementary Figure S3**). This high degree of synteny indicated strong evolutionary constraint on chloroplast genome organization in this subfamily. Although no large-scale rearrangements or inversions were detected, we identified four discrete regions (5,000–10,000, 30,000–35,000, 50,000–55,000, and 125,700–130,000 bp) exhibiting elevated sequence variation. These variable hotspots may represent evolutionarily labile regions that tolerate high nucleotide substitution rates and maintain overall genome structure.

### Phylogenetic relationships

**Figure 4:**
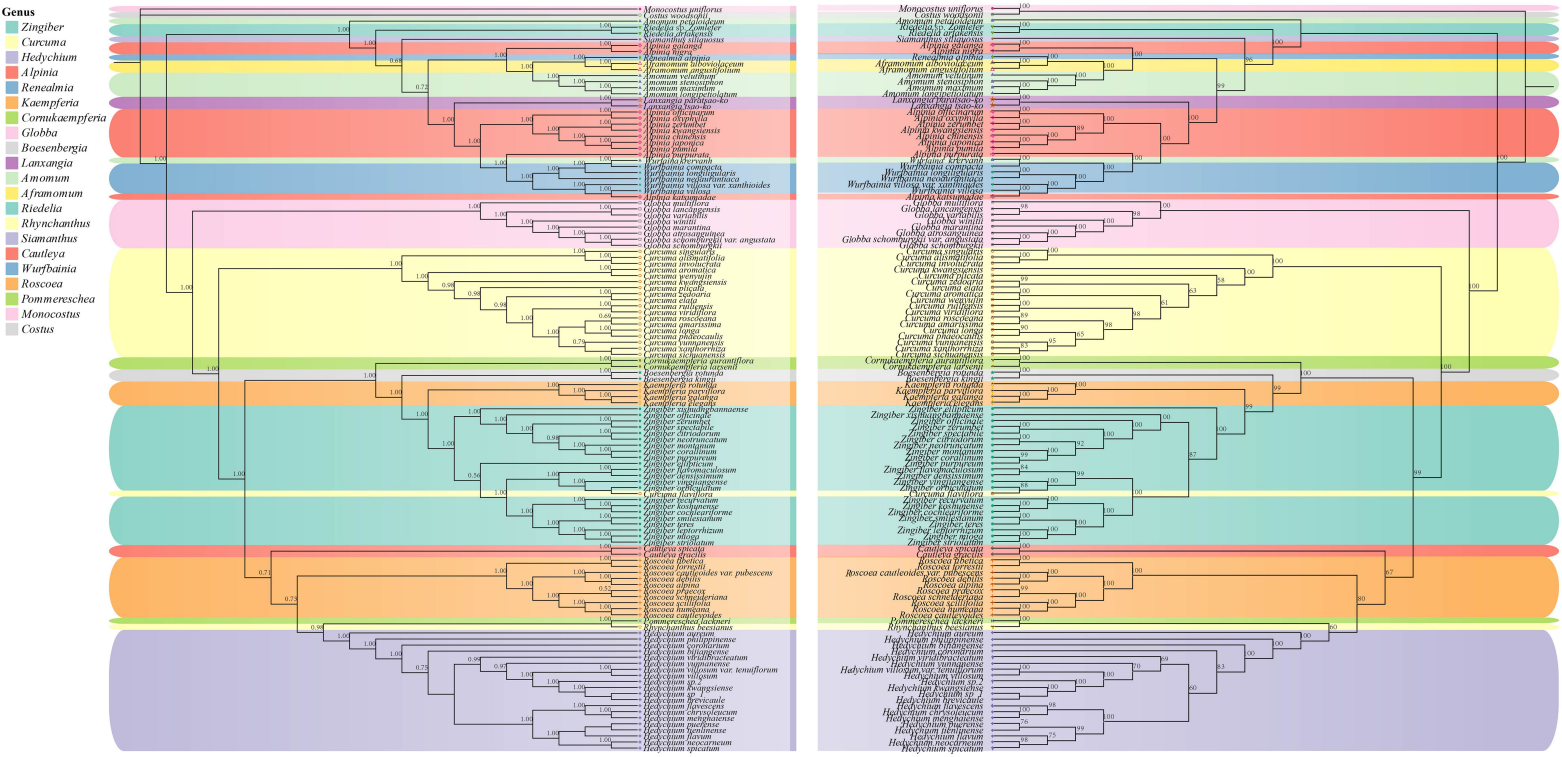
Phylogenetic relationships of 121 Zingiberaceae species taxa inferred from BI, and ML analyses of PCGs. *Costus woodsonii* and *Monocostus uniflorus* were used as the outgroup. BI tree on the left and ML tree on the right. Numbers above nodes are supported values with Bayesian posterior probabilities (PP) values and ML bootstrap values.

Phylogenetic trees were constructed by maximum likelihood (ML) and Bayesian inference (BI) methods for the 123 species. The results demonstrated a high degree of consistency in the topologies of the phylogenetic trees generated by these two methods (**Figure 4**). Our phylogenetic findings provided robust support at the genus level for *Globba* L., *Curcuma*, *Roscoea*, *Kaempferia* L., and *Zingiber* (with posterior probability > 0.9 for the BI tree and bootstrap value > 90 for the ML tree). Nonetheless, we observed some differences in the topology of certain species within the genera *Zingiber* and *Curcuma*. The topological structures of the BI and ML trees consistently classified the species into two subfamilies: Alpinioideae and Zingiberoideae, with strong support (posterior probability = 1.00 for the BI tree and bootstrap value = 100% for the ML tree). The two subfamilies were further subdivided into five clades. the *Riedelia-Siamanthus-Alpinia-Renealm- ia-Aframomum-Amomum-Lanxangia* M. F. Newman & Skornick*.-Wurfbainia* clade (hereafter called the RSARALWA clade) comprised 30 species, and the *Cautleya-Roscoea-Pommereschea* Wittm.*-Rhynchanthus* Hook. f.*-Hedychium* J. Koenig clade (hereafter called the CRPRH clade) consisted of 25 species. Additionally, the *Globba* clade (hereafter called the *Globba* clade) comprised 8 species, and the *Curcuma* clade (hereafter called the *Curcuma* clade) consisted of 18 species, excluding *C. flaviflora*, which clustered with *Zingiber*. The *Roscoea* clade (hereafter called the *Roscoea* clade) contained 10 species, and the *Cornukaempferia* Mood & K. Larsen*-Boesenbergia* Kuntze*-Kaempferia-Zingibe*r clade (hereafter called the CBKZ clade) contained 31 species. Notably, species of the genera *Amomum* and *Alpinia* were divided into three distinct clades in this. Additionally, *C. flaviflora* within the genus *Curcuma* clustered into one clade with the *Zingiber* genus.

Our results indicated that *Aframomum* was a sister clade to the genus *Renealmia*, with strong support (posterior probability = 1.00 for the BI tree and bootstrap value = 100% for the ML tree), and *Cornukaempferia* was most closely related to *Cautleya* and *Boesenbergia*. Furthermore, our results strongly support the monophyletic nature of the genus *Globba*, which appeared as a sister taxon to the genus *Curcuma* (posterior probability = 1.00 for the BI tree and bootstrap value = 100% for the ML tree). Specifically, *G. variabilis* Ridl. and *G. lancangensis* Y. Y. Qian, as well as *G. atrosanguinea* Teijsm. & Binn. and *G. schomburgkii* Hook. f., were identified as sister clades, respectively. *A. velutinum* demonstrated a close relationship with *A. stenosiphon* K.Schum., whereas *A. petaloideum*, while not clustering with other *Amomum* species, appeared to be closest to the genus *Riedelia*. Notably, we established for the first time the close relationship between *Riedelia* and *Siamanthus*. Conversely, *Z. citriodorum* formed a sister clade relationship with *Z. neotruncatum* T. L. Wu & al.

### Estimation of divergence times and biogeographical reconstruction

**Figure 5:**
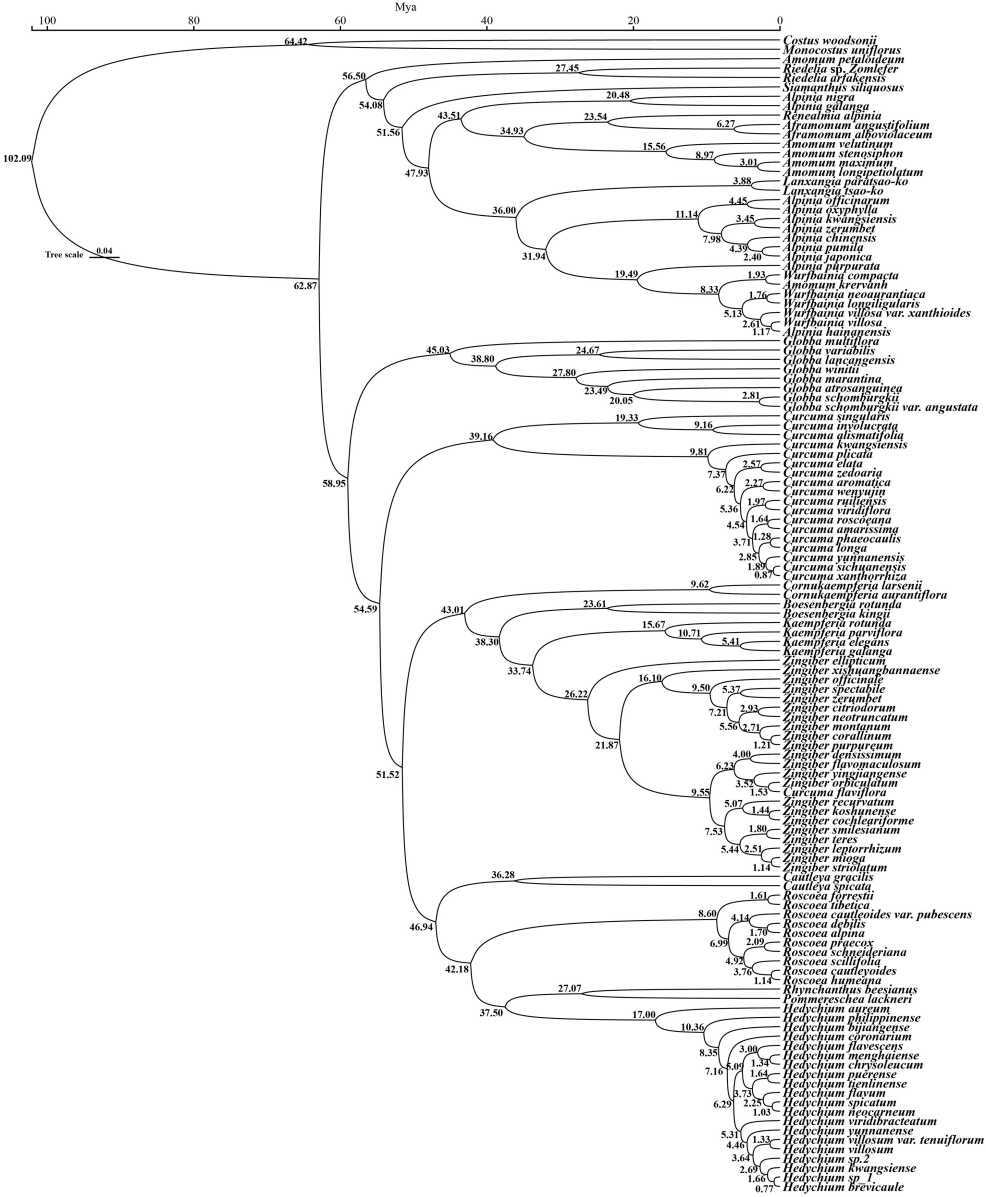
The divergence time of 121 Zingiberaceae species and 2 outgroups based on 78 PCGs. The green boxes indicate 95% confidence intervals, values are shown next to nodes represent divergence times.

**Figure 6:**
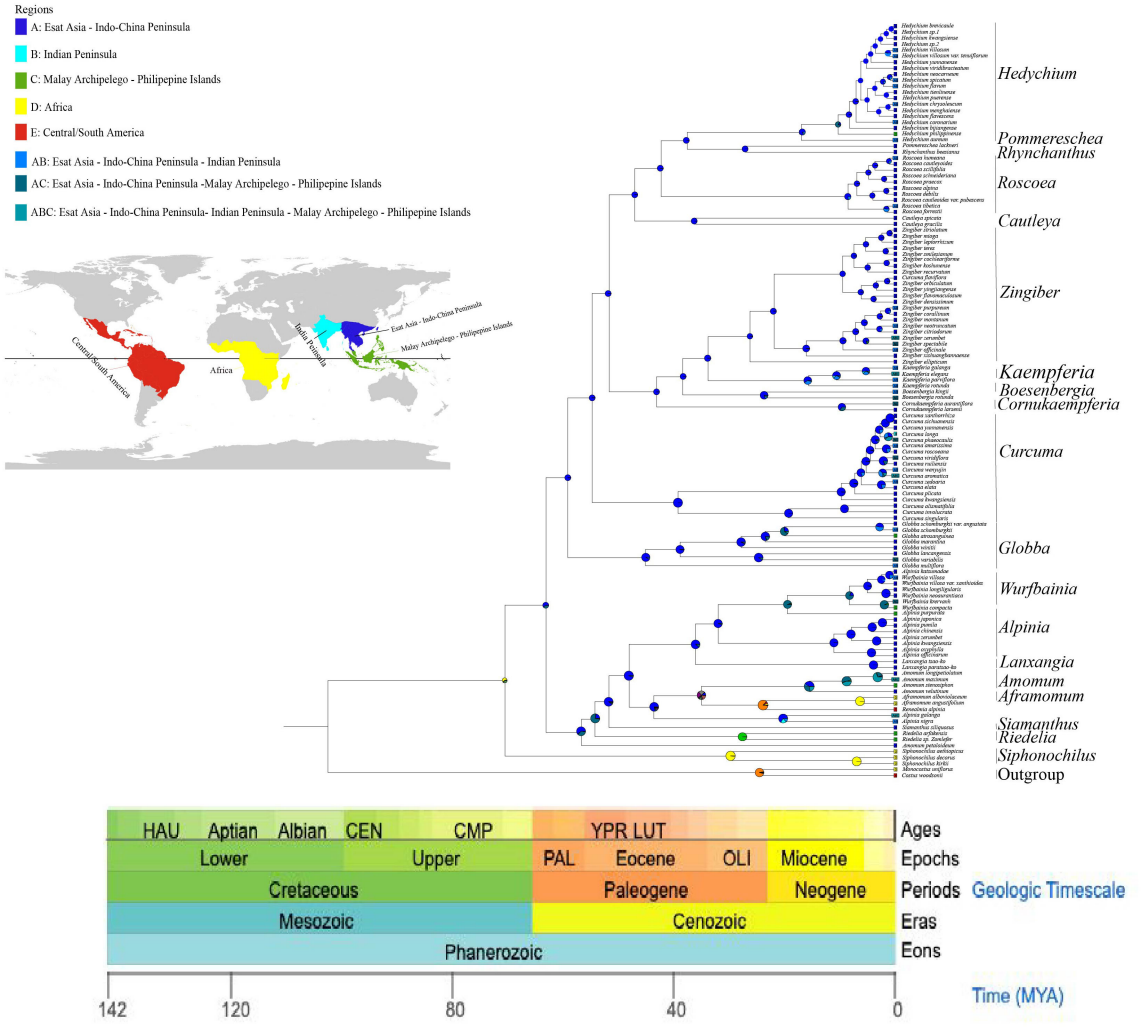
Estimating ancestral ranges of Zingiberaceae species using BioGeoBEARS and DEC model.

On the basis of three fossil-calibrated phylogenies (**Figure 5**), we estimated that the most recent common ancestor (MRCA) of Zingiberaceae and Costaceae existed in the late Cretaceous at ~102.09 million years ago (mya; middle Cretaceous). The Zingiberaceae family initially diverged into two subfamilies of Alpinioideae and Zingiberoideae approximately 62.87 mya (early Palaeocene). Within the Zingiberoideae subfamily, *Globba* diverged from other Zingiberoideae species around 58.95 mya (late Palaeocene), followed by *Curcuma* at about 54.59 mya (from Palaeocene to early Eocene), and *Roscoea*, along with the remaining genera of the RPRH clade, around 46.94 mya (middle Eocene). *Riedelia* diverged from *Siamanthus* at about 54.08 mya (from Palaeocene to early Eocene), *Aframomum* and *Renealmia* diverged around 23.54 mya (from Oligocene to early Miocene), and *Zingiber* diverged from *Kaempferia* at about 33.74 mya (Oligocene), with their common ancestor diverging from *Boesenbergia* around 38.30 mya (Eocene). *Boesenbergia* diverged from *Cornukaempferia* at about 43.01 mya (middle Eocene), and their common ancestor diverged from *Cautleya* around 46.94 mya (Eocene). *Alpinia* diverged from *Lanxangia* at about 36 mya (late Eocene), followed by divergence from *Wurfbainia* around 19.49 mya (Miocene). *Rhynchanthus* diverged from *Pommereschea* at about 27.07 mya (Oligocene), and the common ancestor of the two genus diverged from *Hedychium* at about 37.05 mya (Eocene). The corrected Akaike information criterion (AIC) model selection supported the DEG model, despite the BAYAREALIKE model providing similar results in model selection and ancestral area reconstruction (**Supplementary Figure S4**; **Supplementary 8**). Estimates of ancestral ranges suggested that the Zingiberaceae species probably originated in Africa (Africa at Zingiberoideae vs. Siphonochiloideae divergence node: P_D_= 60.01%), expanded into East Asia and Central and Southern peninsulas, dispersed into the Indian subcontinent, and ultimately to the islands of the Pacific and Indian Oceans (**Figure 6**).

### Characterization of substitution rates and positive selection analyses

**Figure 7:**
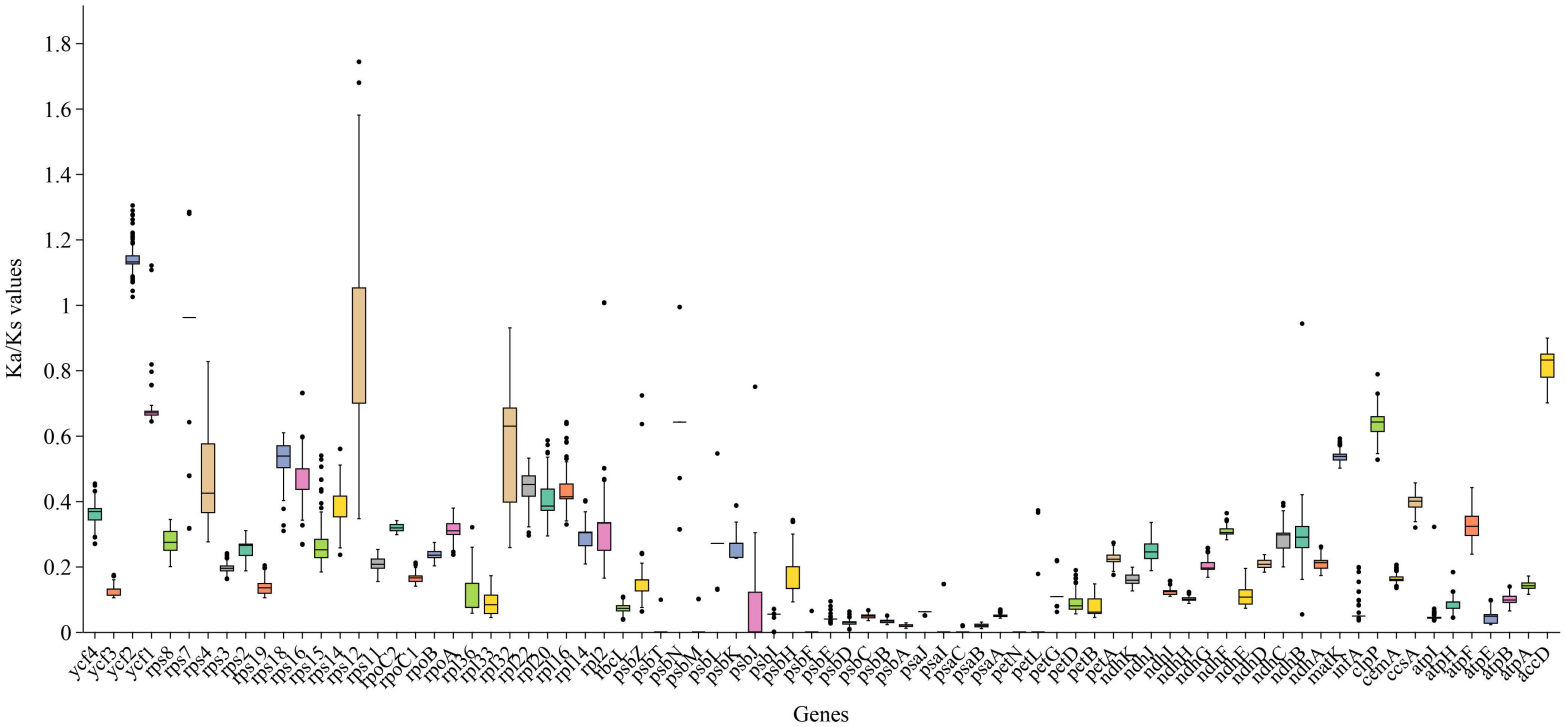
Boxplots of Ka/Ks for the 78 chloroplast PCGs of the 121 Zingiberaceae chloroplast genomes examined. The average Ka/Ks were greatest in *ycf2*.

The Ka/Ks ratio is often used to measure the evolutionary pressure on genes. Analysis of Ka/Ks ratios for 78 PCGs across 121 Zingiberaceae chloroplast genomes revealed distinct evolutionary patterns (**Figure 7**; **Supplementary Figure S5**). The results revealed that the majority of the genes in the Zingiberaceae species exhibited Ka/Ks ratios lower than 1 compared with *M. uniflorus* (**Figure 7**; **Supplementary Figure S5**). This observation indicated that the majority of PCGs in the Zingiberaceae chloroplast genomes have undergone remarkable purifying selection during evolution. Notably, the *ycf2* gene displayed the highest average Ka/Ks value, and all *ycf2* genes across 121 Zingiberaceae species exhibited values exceeding 1. This result implied that the *ycf2* gene might have evolved more rapidly than the other PCGs within the Zingiberaceae chloroplast genomes (**Figure 7**). Additionally, the Ka/Ks ratios of *rps12* were higher than 1 in some species, such as *A. krervanh*, *W. compacta* (Sol. ex Maton) Škorničk. & A.D.Poulsen, and *W. villosa* (Lour.) Škorničk. & A.D.Poulsen, indicating that the *rps12* gene was also under positive selection (**Supplementary 6**). By contrast, the *psa* and *psb* genes exhibited extremely low Ka and Ka/Ks values, with Ka values approaching 0, indicating that these genes were functionally conserved and lacked non-synonymous mutations.

Positive selection signals are commonly considered imprints of species adapting to their environment. Therefore, 6 *Roscoea* species were selected as the foreground for positive selection analysis, utilizing genomic data from 121 Zingiberaceae chloroplast genomes. The results based on the branch-site model analysis revealed the presence of two PCGs, *matK* and *ndhB*, in *Roscoea* species (P < 0.05; **Table 3**).

**Table 3:** Positively selected genes and sites detected in the chloroplast genomes of *Roscoea* genus.

Gene ID	-ln L (Alternative model)	-ln L (Null model)	LRT	P-value	BEB sites
*matK*	-4053.374987	-4057.501107	8.25224	0.004070174	16 N; 225 F; 243 H; 313 Q; 399 T
*ndhB*	-2444.644374	-2441.330465	6.627818	0.010039818	285 L

In positive sites, integers represent the position of the site, letters represent the type of amino acid, and decimals represent the posterior probability

## Discussion

### Comparative analysis of chloroplast genomes

The size, structure, and gene order of the complete chloroplast genomes of the 11 newly assembled Zingiberaceae species were consistent with those of other published species within the Zingiberaceae family, indicating the conservation of chloroplast genomes in this taxonomic group (Wu et al., 2017; Li et al., 2020b; Zhang et al., 2021). No significant expansion or contraction was observed in the chloroplast genomes of Zingiberaceae species throughout the diversification process. Four PCG regions (*atpH*, *rpl32*, *ndhA*, and *ycf1*) and one intergenic region (*psaC-ndhE*) have been identified as highly mutated hotspot regions in Zingiberaceae species. These highly mutated hotspot genes and intergenic regions may serve as valuable molecular markers for phylogeographic and population genetic studies within this family. The RSCU value is a metric indicating the uneven usage of synonymous and non-synonymous codons within a coding sequence. An RSCU ratio <1.00 indicates a lower-than-expected frequency of codon usage, whereas a ratio >1.00 suggests higher-than-expected usage (Liu et al., 2018). Our study revealed that 30 codons had RSCU > 1, with the strongest codon preference observed for UUA, followed by AGA. Previous studies have shown that genome-wide mutation biases are generally reported to be biased toward AT and away from GC (Zhou et al., 2014; Pouyet et al., 2016; Gao et al., 2019). This bias is fixed to some degree by selection, since most but not all changes effectively observed during evolution decrease the genomic GC content (Bohlin et al., 2018; Ely, 2021; Mahajan and Agashe, 2022). Amino acids with A/T at the second positions of their codons (Asn, Asp, Gln, Glu, His, Ile, Leu, Lys, Met, Phe, Tyr, and Val) are on average less hydrophobic (Alff-Steinberger, 1969). This observation may be related to the fact that degeneracy at the third codon position is determined by base pair stability at the second position (Lehmann and Libchaber, 2008; Ye and Lehmann, 2022) and hints at a connection between protein folding, codon-anticodon pairing and the dependence of codon usage on genomic GC content. This codon usage pattern was similar to those reported for other chloroplast genomes (Wu et al., 2017), potentially influenced by a compositional bias toward a high proportion of A/T, which may be attributed to the functions of amino acids or peptide structures, facilitating the mitigation of transcriptional errors (Li et al., 2016; Gao et al., 2019). This phenomenon suggested that the evolution of stable chloroplast genomes not only safeguards crucial chloroplast genes from deleterious mutations but also enhances adaptation to selection pressure (Gao et al., 2019; Zhang et al., 2021; Zhang et al., 2025). Furthermore, all PCGs within the chloroplast genomes of Zingiberaceae were found to fall below the expected ENc curve, indicating that natural selection significantly influenced codon usage preferences (Wang et al., 2016).

### Phylogenetic relationships

The topological structures of the BI and ML trees were consistent and divided into two subfamilies: Alpinioideae and Zingiberoideae. These subfamilies could be further divided into five clades: RSARAALW clade, CRPRH clade, *Globba* clade, *Curcuma* clade, and CBKZ clade. The RSARAALW clade constituted the subfamily Alpinioideae, whereas the remaining clades together constituted the subfamily Zingiberoideae. In this study, the *Globba* clade of the subfamily Zingiberoideae shared a recent common ancestor with the *Curcuma* clade, which contradicted the results of a previous study by Li et al (Li et al., 2020b), who showed that the *Curcuma* clade shared a recent common ancestor with *Hedychium*. This discrepancy may be due to the inclusion of more species in the present study (a total of 121 species in 19 genera), whereas only 8 genera were included in the study by Li et al (Li et al., 2020b). Furthermore, Li et al.’s results placed *Hedychium* within *Curcuma*, which may be incorrect. The CBKZ clade formed a sister clade relationship with the CRPRH clade, which was inconsistent with previous studies. Kress et al (Kress et al., 2002). demonstrated that *Cautleya-Rhynchanthus-Pommereschea-Roscoea* and *Boesenbergia-Kaempferia-Zingibe-Hedych-*.

*ium* constituted a sister clade relationship. Yang et al (Ivanova et al., 2017). supported the conclusion that *Hedychium* was closely related to *Cautleya*. Their research results showed that there were conflicts between the ML tree and BI tree regarding *Pommereschea*-*Rhynchanthus* and *Roscoea* and *Cautleya*, and the support rates of ML and BI were only 50 and 0.8, respectively. Therefore, our results further support the closer phylogenetic relationship between *Cautleya* and *Roscoea*. Overall, our results supported the hypothesis that *Hedychium* was most closely related to *Pommereschea-Rhynchanthus* than to *Cautleya* (posterior probability = 0.98 for the BI tree and bootstrap value = 95% for the ML tree). *Zingiber* was identified as a sister to *Kaempferia* with strong support (**Figure 4**), consistent with previous studies (Kanlayanapaphon and Newman, 2003; Li et al., 2020a; Yang et al., 2022). *Z. ellipticum* was the first lineage to split from *Zingiber* in this study, whereas the remaining *Zingiber* species formed a monophyletic clade with strong support, consistent with previous studies (Li et al., 2020a; Li et al., 2021).

Overall, the phylogenetic relationships within the subfamily Zingiberoideae were elucidated. However, the phylogenetic relationships within the subfamily Alpinioideae were highly intricate, exemplified by genera such as *Alpinia*, *Amomum*, and *Curcuma*. In our study, *Amomum* was categorized into three distinct clades, with *A. velutinum*, *A. stenosiphon*, *A. longipetiolatum* Merr., and *A. maximum* Roxb. forming a cohesive clade. By contrast, *A. petaloideum* and *A. krervanh* each constituted a separate clade. A prior investigation utilizing *matK* and *nrITS* data identified 9 clades within *Amomum*, suggesting its non-monophyletic nature (Larsen et al., 1998). Our findings corroborated and supported this conclusion. Similarly, *Curcuma* displayed 2 clades, *Curcuma* I and *Curcuma* II, where *Curcuma* II comprised solely *C. flaviflora*, whereas the remaining *Curcuma* species were grouped in *Curcuma* I. The clustering of *C. flaviflora* with *Zingiber* aligned with previous research (Cui et al., 2019; Gui et al., 2020; Liang et al., 2020), likely due to overlapping distribution regions and widespread introgressive hybridization (Liang et al., 2020). Taxonomically, *Alpinia* has consistently presented difficulties due to its complexity (Li et al., 2016; Wang et al., 2016; Zhang et al., 2025). The results of ML and BI phylogenetic trees further validate prior reports on the phylogenetic relationships of Zingiberaceae (Larsen et al., 1998; Kress et al., 2002; Li et al., 2020a; Zhang et al., 2021). In conclusion, our results supported that the genus *Alpinia* was the highly polyphyletic genus, and *Alpinia katsumadae* K. Schum. was likely to be clade VII of the genus. Our present findings and the results of Boer et al. (Boer et al. 2018), Rangsiruji et al. (Rangsiruji et al., 2020) and Kress et al. (Kress et al., 2005) on *Alpinia* revealed that the congruence between the major clades of the genus *Alpinia* proved that the genus is polyphyletic, and *A. katsumadae* may be clade VII. The phylogenetic relationships of Zingiberaceae, whose species are extremely diverse and widely distributed, remain unresolved due to the lack of chloroplast genomic data. Thus, further studies and specific sampling will be needed to elucidate the evolutionary complexity of Zingiberaceae which includes other polyphyletic genera.

### Estimation of divergence times and biogeographical reconstruction

Our divergence time estimates revealed three major periods of species radiation in Zingiberaceae that correlated with significant paleoclimatic events. Our estimates indicate that key diversification events (such as the *Roscoea*–*Cautleya* diversification event approximately 46.94 Mya; the *Roscoea*–*Hedychium* divergence event approximately 42.18 Mya) align temporally with the early stages of the Tibetan Plateau uplift (~50–40 Mya). The subsequent diversification pulse occurred around 10 mya, coinciding with the rapid uplift of the Tibetan Plateau (10.5–8 mya) that elevated the region by approximately 1 km and caused an 8 °C decline in temperature (Kanlayanapaphon and Newman, 2003; Chen et al., 2019; Harrison et al., 1992; Molnar et al., 1993; Zhisheng et al., 2001; Li et al., 2020a; Li et al., 2021; Yang et al., 2022; Jiang et al., 2023). This dramatic environmental change appears to have triggered the first major divergence event in Zingiberoideae, including the emergence of the high-altitude genus *Roscoea* at ~8.60 mya. A second radiation event occurred around 5 mya, involving multiple genera (e.g., *Zingiber*, *Hedychium*, and *Curcuma*), which corresponded to the Late Miocene cooling period (7–5.4 Mya) marked by declining atmospheric CO_2_ levels (Tzanova et al., 2015; Zhang et al., 2024). These temporal correlations strongly suggested that the Tibetan Plateau uplift and subsequent Late Miocene cooling acted as key drivers of Zingiberaceae diversification through the creation of new ecological niches and selection pressures. we also note that other regional tectonic events (such as the retreat of the Neotethys Ocean) may have contributed to changes in the biogeographic patterns of the Asian continent. Therefore, we suggest that the Tibetan Plateau uplift may be a major but not the sole driving factor.

This study provided a robust phylogenetic framework for the study of the evolutionary and geographic history of Zingiberaceae species. We reconstructed the divergence times and distribution locations of Zingiberaceae species using the BIOGEOBEARS model in combination with fossil evidence (**Supplementary 8**). The DEC model received overwhelming support with an AICc weight of ≈1.0 (99.99%), significantly outperforming all other models (including BAYAREALIKE: weight was <0.02). Despite BAYAREALIKE having a moderately low AICc (459.20), DEC’s combination of lower AICc (447.54) and higher weight made it the optimal choice. DEC’s parameters (d = 0.636, e = 1.303) suggest that the taxon’s biogeographic history was shaped by moderate dispersal rates coupled with significant extinction events, consistent with known paleoclimatic fluctuations (e.g., Quaternary glaciations). The negligible jump dispersal parameter (j ≈ 0 in DEC) indicates that long-distance dispersal was not a dominant factor, aligning with the taxon’s limited vagility. The model predictions supported a possible African origin for Zingiberaceae, which was consistent with previous findings (Herbert et al., 2016). Africa and tropical America are recognized as the ancestral distribution of the common progenitor of Zingiberaceae and Costaceae, with supporting evidence suggesting their origin in Gondwana during the mid-Cretaceous era and then diverging into separate lineages prior to the final separation of northern Western Gondwana (South America and Africa) (Zhao et al., 2022). This phenomenon, coupled with evidence of Cretaceous-era tropical conditions in northern Africa, suggested that Zingiberaceae may have originated in northern Africa and then spread to other regions. After their divergence from the subfamily Siphonochiloideae, the two subfamilies diversified synchronously from the Paleocene to the Oligocene (**Figure 6**). The ancestral range of Zingiberoideae and Alpinoideae was likely to be in the region of East Asia-Central–South China Peninsulas (P_A_=67.21%). This finding was consistent with the results of Zhao et al (Kress and Specht, 2006), which also indicated that the ancestor distribution of Zingiberoideae and Alpinoideae was Indo-Burma. The ancestor of Zingiberoideae likely originated from East Asia-Central and South China Peninsula (P_A_=99.18%; **Figure 6**), whereas the ancestor of Alpinoideae might have originated from East Asia-Central–South China Peninsula (P_A_=64.23%) and Indian Peninsula (P_B_=33.54%). Our results revealed that Zingiberaceae species underwent rapid radial evolution from 10 Mya to 5 Mya, which was consistent with the findings of Zhang et al. Their results suggested that the dispersal rate of Zingiberaceae among Malay Peninsula, Central South Peninsula, and India increased rapidly after 10 mya and peaked after 5 mya.

### Positive selection analysis

The Zingiberaceae family exhibits a vast distribution across diverse latitudinal and altitudinal ranges, thereby encompassing a rich array of ecological environments. This diversity enhances the observed ecological variability within Zingiberaceae. Our study revealed that the *ycf2* gene underwent positive selection in Zingiberaceae species, which was consistent with previous studies (Wu et al., 2017). Additionally, similar findings were observed in the analysis of other species’ chloroplast genes (Kress and Specht, 2006), indicating that chloroplast genes may experience varying degrees of selection pressure across different plant species. Zingiberaceae predominantly consists of warm and moisture-dependent plants that thrive in tropical and subtropical regions characterized by high humidity and warmth (Liu, 2003). However, the ecological niche of *Roscoea* sharply differs from that of most low-altitude Zingiberaceae species. *Roscoea* species inhabit high mountainous regions, ranging from 1,800 m to 4,000 m above sea level, making them ideal candidates for comparative genomic studies. We hypothesized that the chloroplast genes of *Roscoea* have undergone intense selective pressure to adapt to high-altitude environments, which are characterized by factors such as increased light exposure, intense radiation, low temperatures, and reduced oxygen levels. Consequently, we selected 6 *Roscoea* species as the foreground and 6 low-altitude distribution species as the background for positive selection analysis. Utilizing the branch-site model, we identified two genes (*matK* and *ndhB*) as PSGs within the 6 high-altitude *Roscoea* species.

Mitochondria and chloroplasts, due to their prokaryotic origin, retain the coding for single intron maturation enzymes. The *matR* and *matK* genes are conserved ORFs present in almost all angiosperms (Liere and Link, 1995; Drescher et al., 2000; Kress and Specht, 2006; Barthet et al., 2020). The *matK* gene is situated within an intron of the chloroplast *trnK* gene, encoding a maturation enzyme crucial for shearing 7 different chloroplast group IIA introns, encompassing transcripts from the *trnK*, *trnA*, *trnI*, *rpl2*, *rps12*, and *atpF* genes (Adams et al., 2002). A prior study demonstrated that the addition of heterologously expressed *matK* protein augments the efficiency of group IIA intron self-scissoring within the second intron of *rps12* (Liere and Link, 1995), marking the first direct evidence of *matK* splicing activity via an *in vitro* assay. The significance of tRNA and protein products derived from these precursor RNAs for plastid translation machinery underscores *matK*’s indispensability for chloroplast function. Without *matK*, the synthesis of chloroplast proteins is impeded, thereby compromising normal chloroplast function. Similarly, the *matR* gene encodes a protein involved in intron splicing, potentially participating in the splicing of family II introns in plant mitogenomes. It is retained as a conserved ORF in the mitogenomes of almost all angiosperms (Barthet et al., 2020). In this study, the *matK* gene of high-altitude taxa underwent positive selection. Chloroplasts play a vital role in plant photosynthesis, and the protein encoded by the *matK* gene contributes to intron splicing within chloroplasts, ensuring the proper functioning of relevant genes. Maintaining photosynthetic efficiency is crucial for plants to acquire energy and promote growth in extreme environments. Although no direct study has confirmed the role of *matK* in aiding plants’ adaptation to the harsh plateau environment, Yu et al (Yu et al., 2023). discovered that the *matR* gene, crucial for plant growth, development, respiration, and stress response, might facilitate the adaptive response of *Rhodiola* species to environmental stresses on the Tibetan Plateau. Therefore, considering the significant environmental changes on the plateau, *Roscoea* plants may need to adjust their gene expression and metabolic pathways to thrive under extreme conditions. Consequently, the *matK* gene might contribute to the adaptive response of *Roscoea* species to environmental stress on plateaus. Additionally, we observed that the *matK* gene showed positive selection in certain aquatic or moisture-loving plants (e.g., *Oryza* L. and *Lupinus* L.) (Zoschke et al., 2010; Jiang et al., 2014), potentially aiding them in adapting to low light levels. In conclusion, the positive selection of the *matK* gene may be related to its involvement in chloroplast translation and photosynthesis.

The NADH dehydrogenase complex plays a crucial role in regulating the balance of ATP and NADPH in the electron transport chain. When plants face stress conditions such as high temperature, drought, and salinity, ATP becomes essential for various processes vital for plant cell adaptation to stress (Burrows et al., 1998; Tang et al., 2021). In terrestrial plants, the NADH dehydrogenase (NDH) complex reduces plastoquinone and drives cyclic electron flow (CEF) around PSI (Yang et al., 2007; Peng et al., 2011). This complex not only generates additional ATP for photosynthesis but also enhances plant resilience under abiotic environmental stressors. Prior research has demonstrated that a salt-tolerant soybean variety exhibits higher CEF activity and ATP accumulation in the light compared with a salt-sensitive variety (Melrose). Additionally, *ndhB* and *ndhH* genes of soybean are significantly up-regulated under salt stress, leading to increased levels of their corresponding proteins (He et al., 2015). These findings suggested that salt-tolerant soybeans expedite CEF under salt stress conditions through a mechanism that modulates NDH expression and boosts ATP accumulation in the presence of light. Simultaneously, the expression of genes associated with Na+ transport are upregulated. This process utilizes energy from ATP to sequester more Na+ into chloroplast vesicles, thereby mitigating damage to the photosynthetic apparatus under salt stress conditions (Peng et al., 2011). Considering that all species within the genus *Roscoea* thrive in high mountainous regions at altitudes ranging from 1800 m to 4000 m, where sunlight is exceptionally intense, any imbalance between energy absorption and utilization can trigger photo-oxidative stress (He et al., 2015). Under such circumstances, photosystems are shielded by photoprotection mechanisms that either increase the rate of energy dissipation or reduce the efficiency of light energy absorption, thereby enhancing the detoxification of oxidative substances (Camarero et al., 2012). Previous studies have demonstrated that the structure and size of the light-harvesting antenna of photosystems in plants may vary in response to short-term stress or long-term adaptation to high radiation (Niyogi, 2000; Huner et al., 2003). Alpine plants must maintain a delicate balance in energy allocation among reduced photosynthesis, enhanced luminescence quenching, and resistance to radiation to thrive in extreme environments. Thus, we propose that positive selection of the *ndhB* gene occurs in *Roscoea* plants to achieve equilibrium between energy uptake and utilization, growth, and defense, enabling adaptation to the intense light environment at high altitudes. Experiments by Endo et al (Drop et al., 2014). indicate that *ndhB* is most likely involved in protecting the photosynthetic apparatus from photodamage. Research by Omelchenko et al (Endo et al., 1999). shows that the loss of the ndh gene is associated with an inability to tolerate light intensity stress. It is well known that plants possess mechanisms to withstand excessive light exposure through cyclic electron transfer. Mutant tobacco plants with a lost function of the ndhB gene demonstrate a greater sensitivity of photosynthesis to the width of stomata opening (Horvath et al., 2000; Omelchenko et al., 2020). However, there are currently no reports on the validation of this gene in other high-altitude plants. Therefore, this hypothesis warrants further experimental validation.

## Materials and methods

### Plant material and raw data acquisition

Leaf tissue samples of *C.* sp*icata* (Sm.) Baker (**Supplementary Figure S1**) were collected in Anlong, Guizhou Province (105.49°E, 25.13°N). The samples were identified by Dr. Tao Yuan from Wuhan University, and the specimen (collection number: Cs_00976) was planted in the herb garden of the same university (Wuhan, China; Tao Yuan). Genomic DNA was extracted using the QIAGEN Genomic Kit, and next-generation sequencing (NGS) was performed on the Illumina HiSeq 4000 platform. All sequencing procedures were carried out by Beijing Biomarker Biotechnology Co. (Beijing, China). Genomic DNA was extracted using the QIAGEN Genomic Kit, and NGS reads were sequenced on the Illumina HiSeq 4000 platform. All sequencing procedures were performed by Beijing Biomarker Biotechnology Co. (Beijing, China). In this study, the chloroplast genomes of 11 Zingiberaceae species were assembled and annotated. Except for *C.* sp*icata*, the raw data of the remaining 10 species were downloaded from the NCBI public database. The SRA number of each species and NCBI accession number of the species used in this study are provided in **Supplementary 7**.

### Assembly and annotation of the chloroplast genomes

The raw sequencing reads underwent adapter sequence removal and low-quality read filtering using BBTools (https://sourceforge.net/projects/bbmap/). The *de novo* assembly of the clean reads was conducted with the default settings of GetOrganelle v1.7.5.3 software (Jin et al., 2020), successfully extracting the chloroplast genomes. The process refers to the research by Zhang et al (Zhang et al., 2025). The resulting chloroplast genomes were polished using clean reads via Pilon v1.23 (Walker et al., 2014). Initial annotation of the chloroplast genomes was performed using Geneious Prime v2024.0.5 (Kearse et al., 2012), referencing *Z. officinale* (NC_044775.1). Annotation errors were manually corrected using Geneious Prime v2024.0.5 (Cui et al., 2019). All tRNA genes were predicted with tRNAscan-SE v2.0 (Chan and Lowe, 2019). Finally, the chloroplast genomes were visualized using CPGVIEW (Greiner et al., 2019).

### Analysis of codon usage and ENc-GC3s

We utilized CodonW v1.4.4 software (Peden, 2000) to analyze RSCU and Geneious Prime v2024.0.5 (Cui et al., 2019) to assess the GC content. An RSCU value greater than 1.00 indicates that a codon is used more frequently than expected, whereas a value less than 1.00 suggests it is used less frequently. Effective number of codons (ENC) plots are commonly employed to examine codon usage patterns within genes. The relationship between ENC and GC3s was visualized using R scripts available at GitHub (https://github.com/taotaoyuan/myscript). Predicted ENC values that fall on or above the expected curve suggest that codon usage is primarily influenced by G + C mutations. Conversely, if natural selection or other factors are at play, predicted ENC values may fall below the expected curve (Wright, 1990).

### Comparative genomic analysis

To compare the chloroplasts of Zingiberaceae species, we selected one species from each genus (*Renealima* and *Siamanthus* without complete chloroplast genomes), resulting in a total of 17 species for comparative genome analysis. *Z. officinale* was used as a reference sequence to enable the LAGAN mode in mVISTA software to generate comparison files (Frazer et al., 2004). The nucleotide diversity (Pi) values and sequence polymorphism of 113 complete Zingiberaceae chloroplasts were evaluated, and the regions of LSC, SSC, and IR were also calculated in DnaSP v5.1 (Librado and Rozas, 2009) with a 200 bp step size and a 600 bp window length. The collinearity regions among chloroplasts of 16 Zingiberoideae species were identified and visualized using Mauve v2.4.0 (Darling et al., 2004) with default parameters.

### Phylogenetic analysis

We utilized complete chloroplast genome sequences from 20 species of Zingiberaceae, representing four genera, for phylogenetic analyses (**Supplementary 5**). Two species from the *Curcuma* genus were included as outgroups. PCGs were extracted using Phylosuite software (Zhang et al., 2020). Gene sequences were aligned with MAFFT v7.394, and poorly aligned regions were removed using the “automated1” parameter in TrimAl v1.4.1 (Capella-Gutierrez et al., 2009). The remaining sequences were concatenated into a single supermatrix using FASconCAT-G v1.0499 (Kuck and Longo, 2014). Phylogenetic analyses were performed using Bayesian inference (BI) and maximum-likelihood (ML) methods. The substitution model and optimal partitioning strategy for each dataset were determined using ModelFinder v2017 (Kalyaanamoorthy et al., 2017) based on the Bayesian Information Criterion (BIC). ML analyses were conducted with IQ-TREE v2.0.7 (Nguyen et al., 2015) under the GTRGAMMA model, employing 2000 bootstrap replicates. For BI, we used MrBayes v3.2.2 (Ronquist et al., 2012), running two independent analyses with 2 × 10^6 generations, sampling every 100 generations. Each method was repeated twice to ensure reliability. A convergence threshold of an average standard deviation of split frequencies below 0.01 indicated credible results. After discarding the first 25% of samples as burn-in, posterior probability values were calculated to generate a consensus tree. We employed the MCMCTree package within PAML v4.9j (Yang, 2007) to estimate divergence times using the following parameters: 1 million generations with sampling every 10 generations after an initial burn-in of 100,000 iterations. We obtained three fossil calibration points from the Timetree5 website (http://www.timetree.org/) and published literature, and specific information is provided in **Table 4**. To ensure the reliability of our results, we conducted two independent MCMC analyses to confirm convergence. Finally, we visualized phylogenetic relationships using the ChiPlot online tool (Xie et al., 2023).

**Table 4:** Divergence times based on fossil calibration.

Taxa	Reference time (Mya) Midian (95% HPD)
Zingiberaceae-Costaceae	104.99 (99-109)
Costaceae crown	70.65 (40.18-112.45)
Crown of Zingiberaceae (Divergence between Siphonochiloideae and other ginger subfamilies)	68.41 (65.05–81.78)
Zingiberaceae crown	74.43 (65.19-105.92)
Divergence between Zingiberoideae and Alpinoideae	54.12 (43.05–65.72)
Crown of Zingiberoideae	43.99 (33.69–54.76)
Crown of Alpinoideae	41.12 (30.53–52.36)
*Cautleya*-*Roscoea* divergence	32.42 (17.87-49.53)
*Roscoea* split	23.28 (13.20-37.80)

95%HPD is 95% highest posterior density.

### Biogeographical reconstruction

To trace the biogeographic history of Zingiberaceae, we used BIOGEOBEARS v.1.1.2 (Matzke, 2013) for ancestral region reconstruction. We included Siphonochiloideae in this analysis and set Costaceae as an outgroup. Time-calibrated phylogenetic trees were obtained from MCMCMCTree software in PAML v4.9j (Yang, 2007) based on the *matK* gene (because Siphonochiloideae lacked a complete chloroplast genome). First, We collected species-level distribution data from the World Checklist of Selected Plant Families (WCSP, 2020), Flora of China (Wu and Larsen, 2001), plant of the world online (https://powo.science.kew.org/), and https://doi.org/10.15468/dl.rhdpmd: GBIF. 29 October 2019). Additionally, we referenced the latest World Flora of Plants Map (Liu et al., 2023) for geographical distribution range coding. We included A, Southern China-Central South Peninsula (including Tibet, China, and Japan); B, Indian Peninsula (including Bangladesh and Sri Lanka); C, Malay Archipelago–Philippine Islands (including the Sunda Shelf, Sulawesi Island, and New Guinea); D, Africa; and E, Central/South America. We tested all six models provided by BIOGEOBEARS, including the LAGRANGE’s DEC model, DIVALIKE, and BAYAREALIKE, along with their respective models with the parameter +J, which facilitated founder-event speciation (Matzke, 2014). In this study, each ancestor was allowed to exist in no more than three regions. This option was chosen because the maximum number of regions in which a Zingiberaceae species can be found is 3 (**Supplementary 7**). The result file from MCMCtree in PAML v4.9j was input to BGB to select the optimal model. The Akaike weight of information criterion (AICc_wt) was used to compare the six models. The likelihood ratio test (LRT) was used to test the null hypothesis, asserting that a model has an equal likelihood value to its +j models. A p-value of <0.05 for LRT indicates the rejection of the null hypothesis. The DEC model performed better, so it was used in this study (**Supplementary 8**). We defined two stratification events based on divergence time and address events, namely, the divergence time nodes at Alpinioideae and Zingiberoideae (62.87 Mya). At the same time, dispersal rates in different geographic regions were considered, as shown in **Supplementary 9**.

### Evolutionary rates and positive selection analysis

To compare the NSR of chloroplasts among the 121 Zingiberaceae species, we used *M. uniflorus* as a reference to calculate the NSR of PCGs in the 121 Zingiberaceae chloroplasts. We extracted 78 chloroplast PCGs using PhyloSuite v1.2.3 (Zhang et al., 2020) and calculated the ratios of synonymous (dS) and nonsynonymous (dN) substitution rates using the KaKs_Calculator v2.0 (Zhang et al., 2006) with the yn00 model. To estimate selection pressure resulting from the plateau environment, 6 *Roscoea* species were defined as foreground branch. The Codeml package in PAML v4.9j software (Yang, 2007) was used to identify potential positive selection genes (PSGs). We performed a Chi-squared test on LRT values to obtain valid P-values (P<0.05) and implemented the BEB method to identify positive selection sites. Positively selected genes were defined as those with p < 0.05 and ω > 1.

## Conclusions

In this study, we presented the chloroplast genomes of 11 Zingiberaceae species, ranging in size from 161,399 bp to 166,207 bp. The 11 *de novo* assembled chloroplast genomes contained 128–132 predicted functional genes, comprising 84–86 PCGs, 35–39 tRNA genes, and 8 rRNA genes. Codon usage analysis revealed the strongest preference for UUA and AGA. ENc-map results indicated that neutral selection might play an important role in shaping codon preferences. The divergence level of the non-coding regions was higher than that of the coding regions. Four PCG regions (*atpH*, *rpl32*, *ndhA*, and *ycf1*) and one intergenic region (p*saC-ndhE*) exhibited significant differences (Pi>0.085), making them suitable DNA markers for species identification and genetic diversity studies in Zingiberoideae plants. Phylogenetic results supported the division of the Zingiberaceae family into two subfamilies: Alpinioideae and Zingiberoideae. Our findings rejected the conclusion that the *Globba* clade had a recent affinity with the *Hedychium* clade and supported the idea that the *Globba* clade had a recent affinity with *Curcuma*. Additionally, our results rejected the notion that *Hedychium* shared a recent common ancestor with *Cautleya* and instead supported the idea that *Hedychium* had a close affinity with *Pommereschea-Rhynchanthus.* Divergence time estimates indicated that Zingiberoideae underwent two rapid divergences, one triggered by the rapid uplift of the Tibetan Plateau around the late Miocene (9.35 mya), and the second around 5.34 mya due to a temperature shift caused by a decline in CO_2_, ultimately leading to rapid divergence. Ancestral range reconstruction results indicated that Zingiberaceae species originated from the African continent during the Cretaceous period, spread to East Asia and Indo-China Peninsula around the Paleogene period, and settled into the Indian Peninsula and the Malay Archipelago region. The results of selection pressure analysis indicated that most PCGs in Zingiberaceae species undergo purifying selection (dN/dS < 1), with the exception of the *ycf2* gene. Furthermore, we identified two PSGs, *matk* and *ndhB*, which may play important roles in the adaptation of *Roscoea* species to high-altitude environments. This study has significantly enriched the genetic resources of Zingiberaceae species and established a robust scientific foundation for the development of molecular markers, taxonomy, and phylogenetic studies. It also provides a theoretical basis for breeding and conservation efforts.

## Data Availability

The datasets presented in this study can be found in online repositories. The names of the repository/repositories and accession number(s) can be found in the article/**Supplementary Material**.
