# Supplementary material for: Molecular phylogenetic relationships based on chloroplast genomes of Zingiberaceae species: Insights into evolution and adaptation to extreme environments
Source: Front Plant Sci. 2025 Sep 24;16:1670568. doi: 10.3389/fpls.2025.1670568 (PMC12504198; doi:10.3389/fpls.2025.1670568)
Supplement: Supplementary file 1 [file DataSheet1.docx]

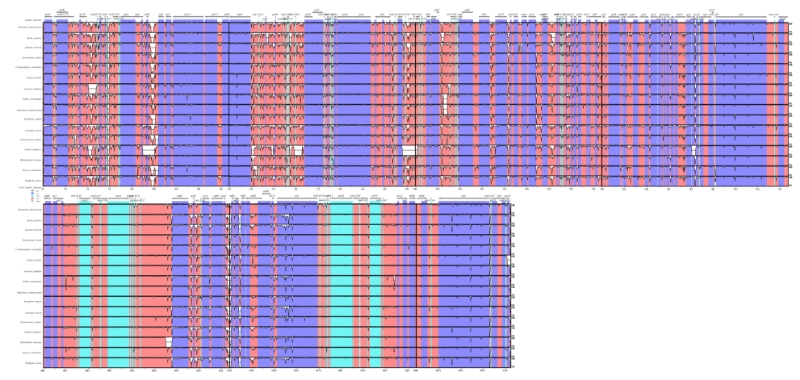


Figure S1. Sequence alignment of the 17 Zingiberoideae CP genomes in mVISTA. The CP genome of *Z. officinale* was used as a reference. Gray arrows and thick black lines above the alignment indicate gene orientation. Purple bars represent exons, sky-blue bars represent transfer RNA (tRNA) and ribosomal RNA (rRNA) and red bars represent noncoding sequences (CNS). The horizontal axis indicates the coordinates within the CP genomes. The vertical scale represents the identity percentage ranging from 50% to 100%. White represents regions with sequence variation among the four species.


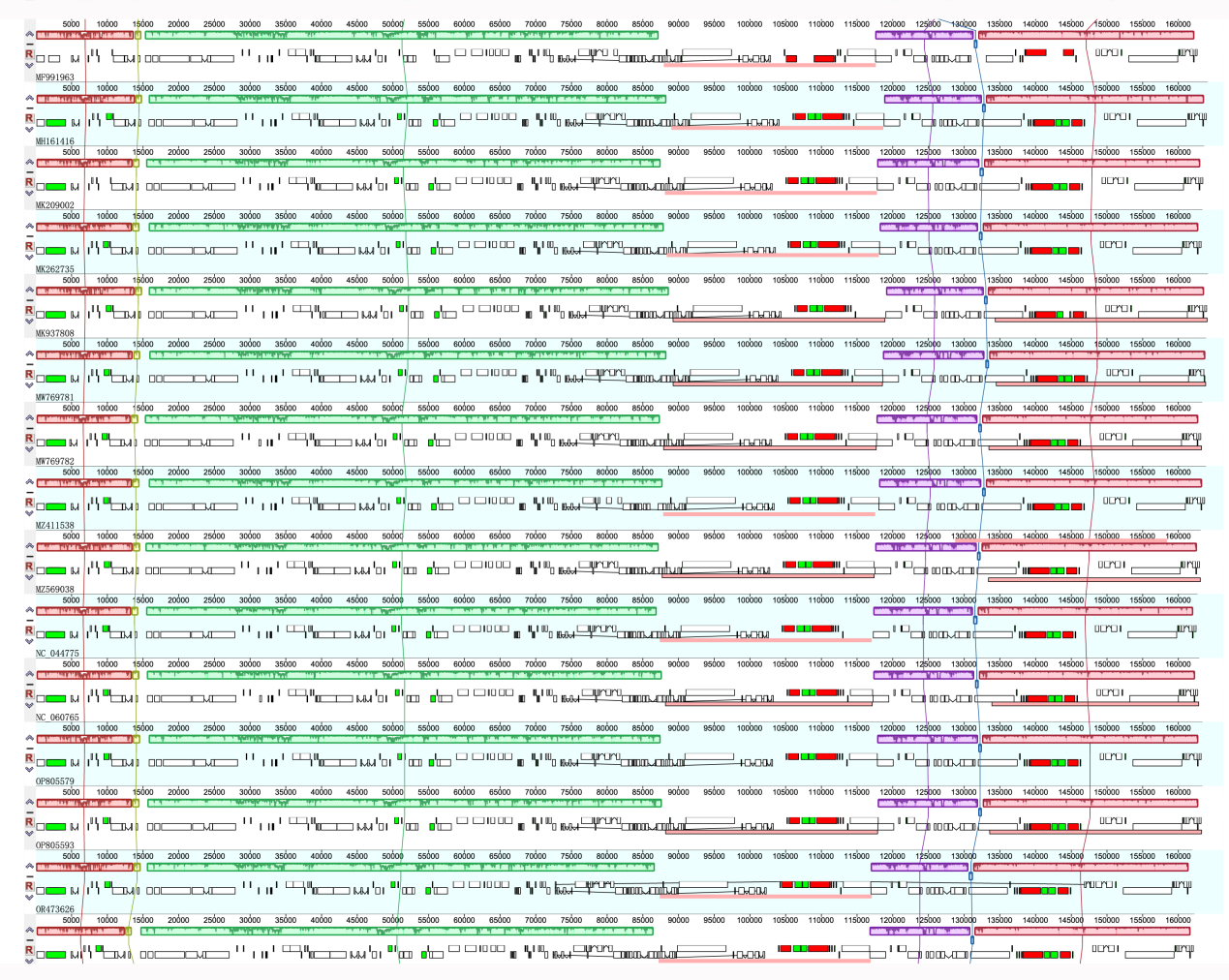


Figure S2. MAUVE alignment of Zingiberoideae species. As a reference, the CP genome of *Z. officinale* was presented at the top. The long squares show genomic similarity, while the lines connecting them represent a covariate association. Each genome’s gene locations are represented by the short squares. CDS is represented by white, tRNA is represented by green, and rRNA is represented by red.


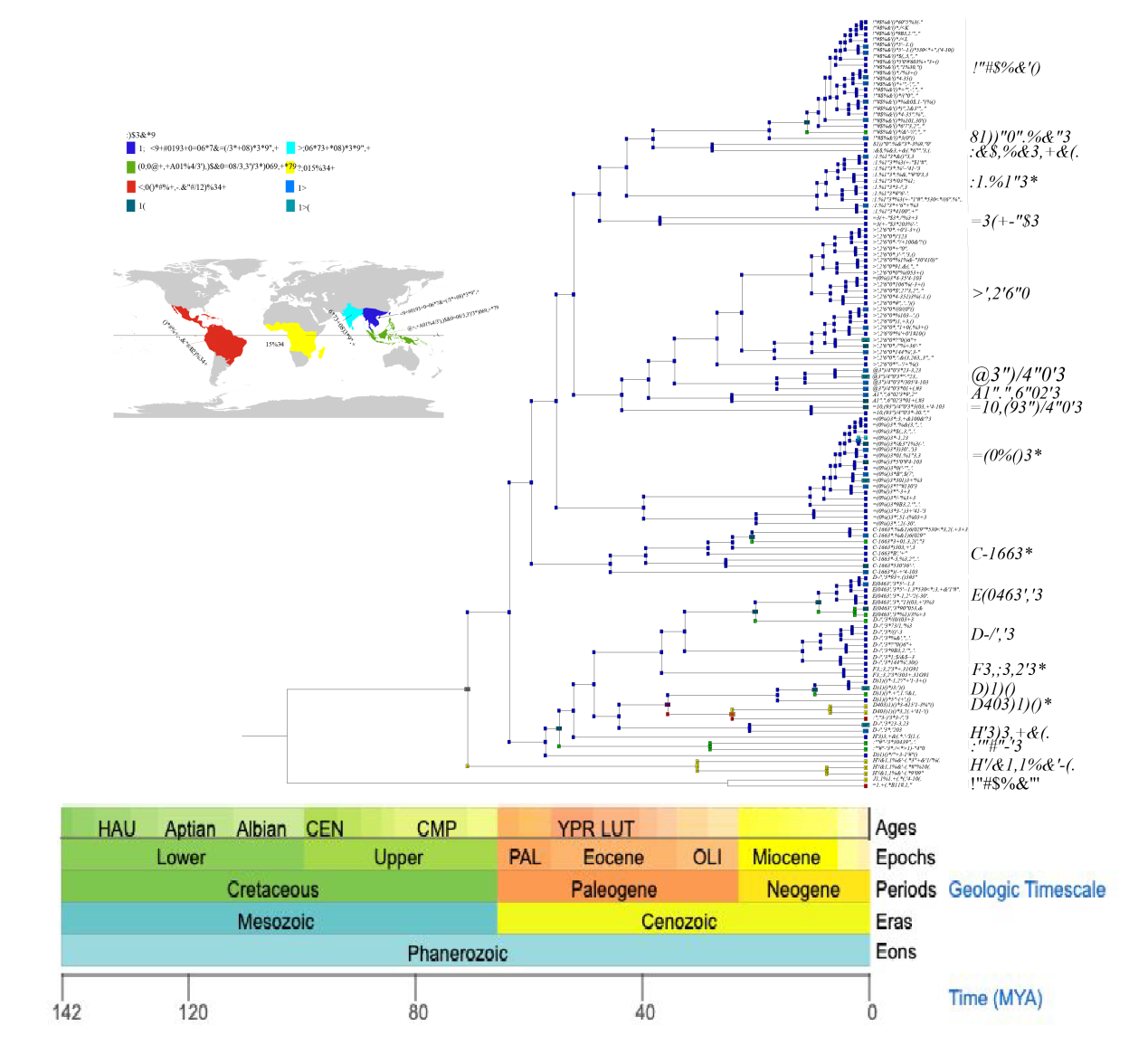


Figure S3. Estimating ancestral ranges of Zingiberaceae species using BioGeoBEARS and BAYAREALIKE model.


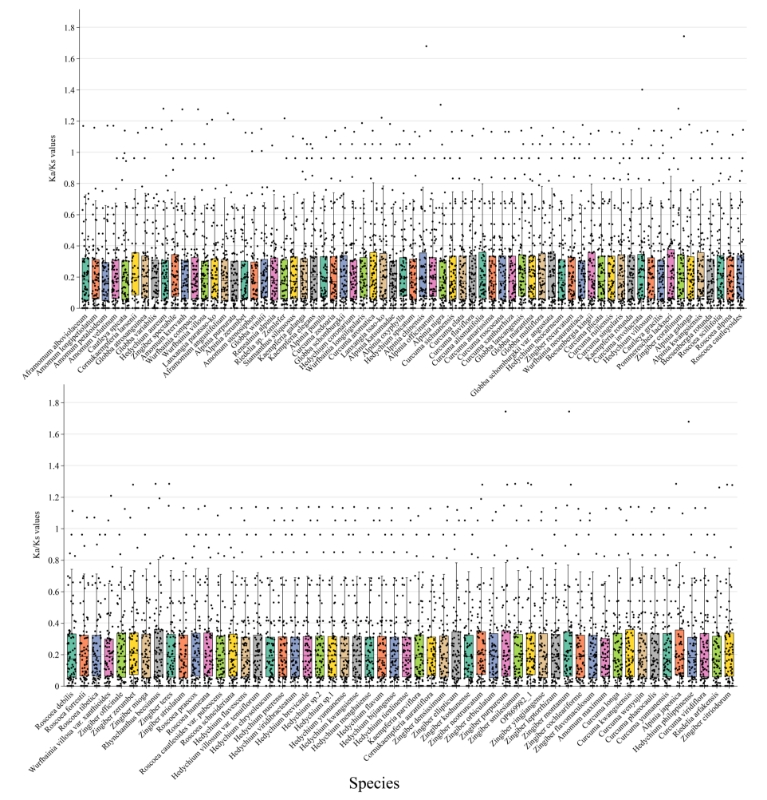


Figure S4. Boxplot of Ka/Ks of the 78 PCGs across the 121 Zingiberaceae CP genomes examined. These data were calculated with the free-ratios model.
